# Comparative Prognostic Accuracy of Objective Nutritional Indices in Critically Ill Patients with Sepsis: A Systematic Review and Meta-Analysis

**DOI:** 10.3390/jcm15103885

**Published:** 2026-05-18

**Authors:** Yan-Wu Yang, Yan Zhang, Tian-Yi Qu, Mei-Ling Ge, Zhi Wan

**Affiliations:** 1 The Emergency Department, West China Hospital, Sichuan University, Chengdu 610041, China; 2 Center of Gerontology and Geriatrics and National Clinical Research Center for Geriatrics, West China Hospital, Sichuan University, Chengdu 610041, China; 3 Laboratory of Cardiac Structure and Function, Institute of Cardiovascular Diseases, West China Hospital, Sichuan University, Chengdu 610041, China

**Keywords:** sepsis, nutritional risk, prognostic assessment, mNUTRIC, prognostic nutritional index (PNI), meta-analysis

## Abstract

**Background**: Nutritional indices are increasingly studied as prognostic tools in sepsis, but their comparative value remains uncertain. We conducted a systematic review and meta-analysis to evaluate associations between major nutritional indices and mortality in adult sepsis. **Methods**: PubMed, Embase, and Web of Science were searched for eligible studies. Pooled odds ratios (ORs), hazard ratios (HRs), and diagnostic accuracy measures were synthesized using random-effects models. Subgroup and sensitivity analyses explored heterogeneity and tested robustness. **Results**: Twenty-two studies comprising 51,769 patients were included. Higher modified Nutrition Risk in the Critically Ill (mNUTRIC) and Nutrition Risk in the Critically Ill (NUTRIC) scores were associated with increased mortality (OR 3.10, 95% CI 1.39–6.89; OR 4.54, 95% CI 2.13–9.66, respectively). In contrast, a higher Prognostic Nutritional Index (PNI) was consistently associated with lower mortality (OR 0.64, 95% CI 0.50–0.83; HR 0.66, 95% CI 0.54–0.81), and a higher Geriatric Nutritional Risk Index (GNRI) was associated with improved survival (HR 0.66, 95% CI 0.44–0.98). Controlling Nutritional Status (CONUT) showed a non-significant trend toward higher mortality (OR 1.83, 95% CI 0.94–3.54). In diagnostic analyses, mNUTRIC demonstrated better discrimination than PNI (AUC 0.84 vs. 0.74). Heterogeneity in mNUTRIC analyses decreased markedly after stratification by mortality endpoint. **Conclusions**: Nutritional indices are prognostically informative in sepsis, but performance is context-dependent. mNUTRIC/NUTRIC show stronger short-term signals in ICU cohorts, likely reflecting illness-severity components, and cross-index comparisons remain indirect due to heterogeneous thresholds and endpoints.

## 1. Introduction

Sepsis remains a leading cause of critical illness and death worldwide. Although defined as life-threatening organ dysfunction resulting from a dysregulated host response to infection, its clinical course is highly heterogeneous, and patients with apparently similar infectious triggers and initial severity may follow very different trajectories after ICU admission [1,2]. Current management emphasizes early recognition, prompt resuscitation, source control, and organ support, yet reliably identifying those at greatest risk of early deterioration or death remains challenging.

Part of this variability may reflect not only the severity of acute organ failure but also differences in underlying host reserve. In this context, nutritional status is unlikely to be a mere background characteristic. Malnutrition, hypoalbuminemia, lymphopenia, weight loss, and impaired metabolic reserve are common in critically ill and septic patients and are consistently associated with higher mortality, longer length of stay, and more complications [3,4,5,6]. These abnormalities may capture a broader state of physiological vulnerability characterized by diminished nutritional reserve, compromised immune function, sustained inflammatory stress, and reduced capacity to withstand critical illness. Thus, in critically ill patients, nutrition-related indices may provide accessible information on host susceptibility and recovery potential, rather than simply indicating inadequate caloric intake [7,8].

Several nutritional tools have been evaluated in sepsis and other critically ill cohorts, but they do not quantify the same dimension of risk. The Nutrition Risk in Critically Ill (NUTRIC) and modified NUTRIC (mNUTRIC) scores integrate acute illness severity and baseline risk, whereas the Prognostic Nutritional Index (PNI), Geriatric Nutritional Risk Index (GNRI), Controlling Nutritional Status (CONUT) score, and other composite scores primarily reflect immunonutritional status and body reserve [3,7]. These tools therefore cannot be assumed to be interchangeable, and their reported prognostic performance has been inconsistent across settings. Existing studies differ in design, mortality endpoints, follow-up duration, cut-off definitions, and analytic methods, and direct head-to-head comparisons are scarce.

A key methodological consideration is that these tools do not quantify the same construct. mNUTRIC and NUTRIC were developed for critically ill populations and incorporate established illness-severity domains (e.g., organ dysfunction and comorbidity burden), whereas PNI, GNRI, and CONUT primarily reflect immunonutritional reserve derived from laboratory markers and body-weight-related parameters. Therefore, apparent differences in prognostic performance should not be interpreted as a definitive hierarchy of ‘nutritional’ tools. Moreover, prognostic meta-analyses require special caution because included studies often report differently adjusted estimates, apply heterogeneous thresholds or categorizations, and define mortality across different time horizons, all of which can materially affect pooled magnitudes and clinical interpretability.

Against this background, we conducted a systematic review and meta-analysis to provide an integrated evaluation of nutritional indices in sepsis. Specifically, we aimed to quantify the association between major nutrition-related scores and mortality, compare their prognostic discrimination using diagnostic performance metrics, and explore whether differences in outcome definitions and follow-up contribute to between-study heterogeneity. Our goal was to clarify the clinical contexts in which these indices may offer meaningful prognostic information, while avoiding overstatement of tools that should be regarded as adjunctive rather than stand-alone predictors.

## 2. Methods

This systematic review and meta-analysis was conducted in accordance with the Preferred Reporting Items for Systematic Reviews and Meta-Analyses (PRISMA) guidelines (checklist provided in Table S1). The review protocol was not prospectively registered (e.g., PROSPERO).

### 2.1. Search Strategy

A comprehensive systematic literature search was performed in PubMed, Embase, and Web of Science from database inception to March 2026. The search strategy combined Medical Subject Headings (MeSH) and free-text terms related to “sepsis,” “septic shock,” “critically ill,” and specific objective nutritional indices, including the PNI, GNRI, CONUT, NUTRIC, and mNUTRIC. During the literature search, we also screened for traditional nutritional screening and assessment tools, including the Nutritional Risk Screening 2002 (NRS-2002), Subjective Global Assessment (SGA), Malnutrition Universal Screening Tool (MUST), Mini Nutritional Assessment (MNA), and the Global Leadership Initiative on Malnutrition (GLIM) criteria. However, quantitative meta-analysis was restricted to objective indices with a sufficient number of available studies (k ≥ 3) reporting comparable prognostic outcomes (detailed syntax in Supplementary Table S2).

### 2.2. Selection Criteria and Data Extraction

Studies were included if they met the following criteria: (1) enrolled adult patients (≥18 years) diagnosed with sepsis or septic shock according to Sepsis-2 or Sepsis-3 criteria; (2) evaluated at least one predefined objective nutritional index (PNI, GNRI, CONUT, NUTRIC, or mNUTRIC); (3) reported clearly defined mortality outcomes, such as 28-day mortality, 30-day mortality, ICU mortality, or in-hospital mortality; and (4) provided sufficient data to extract or calculate odds ratios (ORs) or hazard ratios (HRs) with 95% confidence intervals (CIs). Two independent reviewers performed study screening and data extraction. Studies based on the Medical Information Mart for Intensive Care (MIMIC) databases were carefully cross-checked to minimize the risk of overlapping patient cohorts within specific index analyses.

### 2.3. Quality Assessment

The methodological quality of the included retrospective and prospective cohort studies was assessed using the Newcastle–Ottawa Scale (NOS). Studies were evaluated across three domains: selection of the study groups, comparability of the cohorts, and ascertainment of the outcome of interest. Studies with an NOS score ≥7 were considered to be of high quality.

### 2.4. Statistical Analysis

Pooled ORs and HRs with corresponding 95% CIs were calculated to assess the prognostic association between nutritional indices and sepsis mortality. To account for anticipated clinical and methodological heterogeneity, random-effects models were applied. Specifically, the restricted maximum likelihood (REML) estimator with Hartung–Knapp adjustment was used to provide more robust variance estimation, particularly in analyses involving a small number of studies. Heterogeneity was quantified using the I^2^ statistic, with values > 50% indicating substantial heterogeneity. To explore potential sources of heterogeneity, prespecified subgroup analyses were conducted according to mortality endpoints, including 28/30-day mortality and ICU/in-hospital mortality. For comparative prognostic accuracy, summary receiver operating characteristic (SROC) curves were constructed, and the area under the curve (AUC) was calculated. Fagan nomogram analysis was further performed to evaluate clinical utility and post-test probability. Leave-one-out sensitivity analysis was conducted by sequentially excluding one study at a time to test the robustness of the pooled estimates. Formal publication bias assessment using funnel plots or Egger’s linear regression test was not performed because the number of studies available for each nutritional index was below the recommended threshold of 10 (k < 10), limiting the ability to distinguish true asymmetry from chance.

#### 2.4.1. Effect Estimate Extraction and Harmonization

When both adjusted and unadjusted estimates were available, we preferentially extracted the most fully adjusted effect estimate. For categorical syntheses, we extracted the study-defined ‘worst vs. best’ contrast for each index (e.g., high vs. low mNUTRIC/NUTRIC; higher vs. lower CONUT; lowest vs. highest PNI/GNRI category). Continuous-effect estimates (e.g., per-unit or per-SD increase) were synthesized separately and were not pooled together with categorical contrasts. Because cutoffs and category definitions varied across studies, pooled categorical effects were interpreted as harmonized extreme-category contrasts rather than a single actionable threshold. Prespecified sensitivity analyses restricted to more comparable definitions were conducted where feasible.

#### 2.4.2. Diagnostic Accuracy Synthesis

For prognostic discrimination, we performed a diagnostic accuracy meta-analysis using a hierarchical approach to jointly synthesize sensitivity and specificity and to generate summary receiver operating characteristic (SROC) curves and the area under the curve (AUC). Studies were included in hierarchical pooling only when sufficient information was available to derive 2 × 2 classification data at a reported threshold; otherwise, discrimination metrics were summarized narratively. Given heterogeneous thresholds and outcome definitions, SROC/AUC comparisons across indices were considered indirect.

#### 2.4.3. Study Selection Process and Overlap Checks

Disagreements during screening, data extraction, and quality assessment were resolved by discussion and consensus; when necessary, a third reviewer adjudicated. For database-derived cohorts (e.g., MIMIC), we compared database version, study period, inclusion criteria, and mortality endpoints across reports. Where cohorts were clearly overlapping within a given index analysis, we retained one dataset based on prespecified criteria (largest sample size and/or most fully adjusted model) to avoid double-counting.

All analyses were conducted in R (R Foundation for Statistical Computing, Vienna, Austria; version 4.3.2) using the meta and metafor packages.

## 3. Results

### 3.1. Study Selection and Characteristics

**Figure 1:**
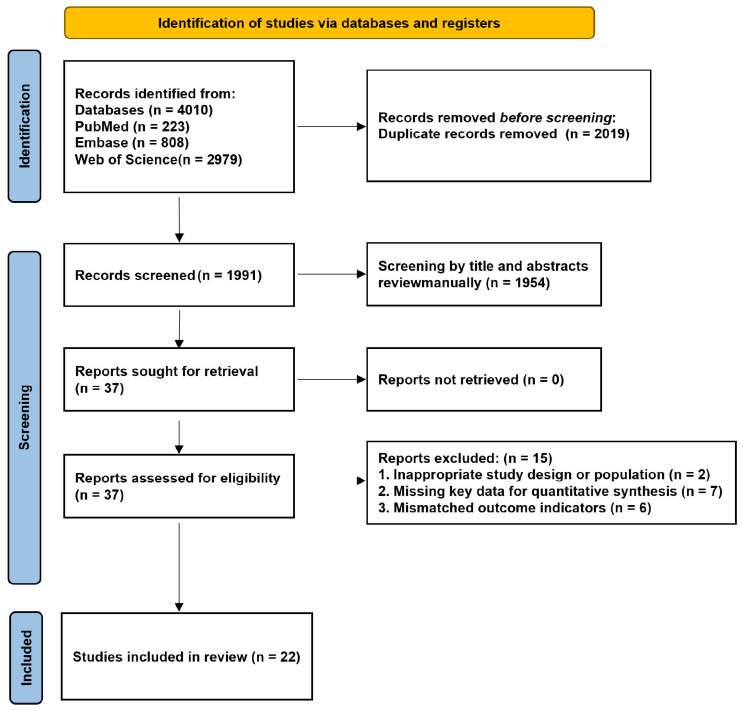
PRISMA flow diagram of study selection.

The study selection process is summarized in Figure 1. A total of 4010 records were identified through database searching, including 223 from PubMed, 808 from Embase, and 2979 from Web of Science. After the removal of 2019 duplicates, 1991 records underwent title and abstract screening, of which 1954 were excluded. The remaining 37 reports were retrieved for full-text assessment.

Following full-text review, 15 reports were excluded because of inappropriate study design or population (n = 2), missing key data for quantitative synthesis (n = 7), or mismatched outcome indicators (n = 6). Ultimately, 22 studies were included in the final systematic review and meta-analysis [9,10,11,12,13,14,15,16,17,18,19,20,21,22,23,24,25,26,27,28,29,30]. Although additional nutritional screening or diagnostic tools, including NRS-2002, SGA, MUST, MNA, and GLIM-based criteria, were identified during the literature search, the available evidence for each was too limited to support quantitative synthesis.

The 22 included studies were published between 2018 and 2026 and comprised a total of 51,769 patients. Of these, 17 studies used a retrospective cohort design, and 5 were prospective. Geographically, the studies were predominantly conducted in China (n = 11), followed by Turkey (n = 4) and Korea (n = 2), with one study each from Japan, Poland, Vietnam, and Taiwan. Sample sizes ranged from 78 to 9763 participants. Across the included studies, the mean or median age of participants generally ranged from 60 to 78 years, and the proportion of male patients ranged from 39.4% to 73.7%. All study populations consisted of patients with sepsis treated in intensive care unit settings. Mortality outcomes were assessed at different time points, including ICU mortality, in-hospital mortality, 28- or 30-day mortality, 90-day mortality, and 1-year mortality. Nutritional status was evaluated using several validated indices, including PNI (n = 9), GNRI (n = 6), mNUTRIC or NUTRIC (n = 8), and CONUT (n = 3). Detailed study characteristics are presented in Table 1. The methodological quality of the included studies was assessed using the Newcastle–Ottawa Scale, and all 22 studies were considered of high quality, with scores ranging from 7 to 9 (Table S3).

### 3.2. Integrative Prognostic Performance of Nutritional Indices

#### 3.2.1. Prognostic Value of mNUTRIC and NUTRIC Scoring Systems

**Figure 2:**
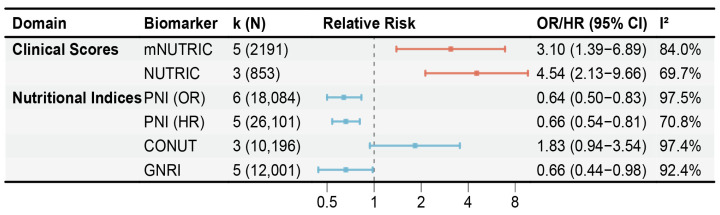
Integrative prognostic landscape of nutrition-related indices and scores in sepsis. Red indicates Clinical Scores and blue indicates Nutritional Indices; horizontal lines show 95% CIs and the dashed line indicates OR/HR = 1.

**Figure 3:**
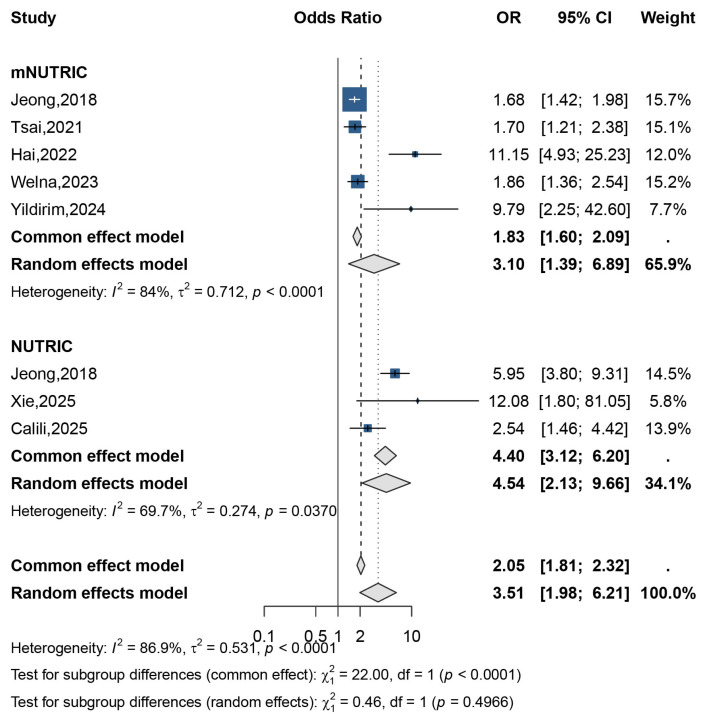
Pooled odds ratios for high vs. low mNUTRIC/NUTRIC in relation to sepsis mortality [9,10,11,14,17,19,26].

The integrated prognostic landscape (Figure 2) reveals divergent predictive strengths among the assessed indices. The pooled analysis identified elevated mNUTRIC and NUTRIC scores as robust predictors of sepsis mortality. Specifically, a high mNUTRIC score was associated with a more than three-fold increase in death risk (OR 3.10, 95% CI 1.39–6.89), while the NUTRIC score showed a similarly strong association (OR 4.54, 95% CI 2.13–9.66). Given the significant heterogeneity observed (I^2^ = 84% and 69.7%, respectively) (Figure 3), a random-effects model was employed for subgroup comparison, which confirmed no statistically significant difference between the mNUTRIC and NUTRIC scoring systems (*p* = 0.4966).

#### 3.2.2. Predictive Performance of PNI, CONUT, and GNRI

**Figure 4:**
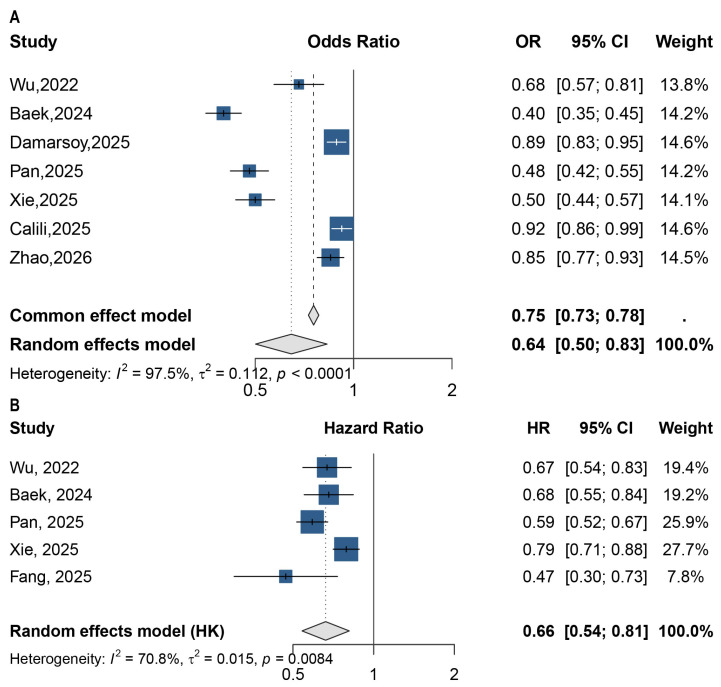
Pooled associations of PNI with sepsis mortality. (**A**) Odds ratio (OR)—based meta-analysis pooling the lowest vs. highest PNI category (categorical contrasts). (**B**) Hazard ratio (HR)—based meta-analysis pooling time-to-event estimates in survival models (as defined in each study) [12,16,19,20,21,23,27,30].

**Figure 5:**
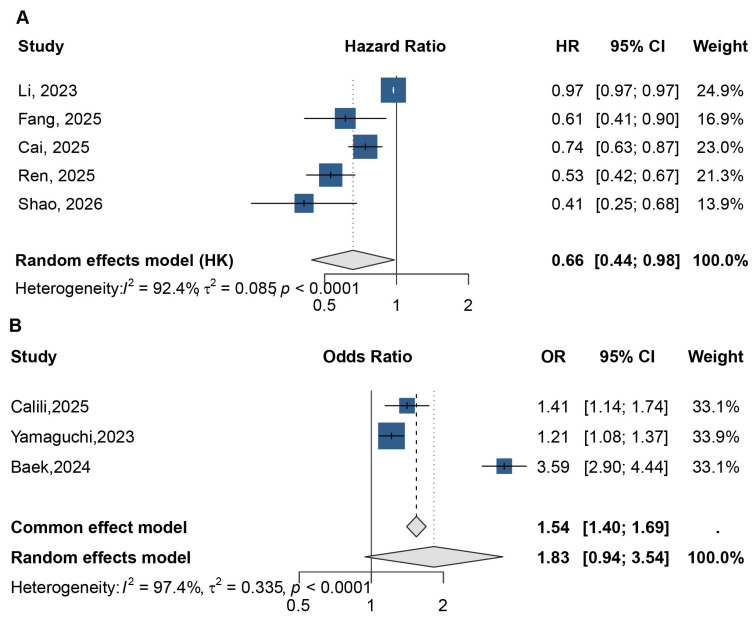
Forest plots of pooled associations for GNRI and CONUT in relation to sepsis mortality: (**A**) pooled hazard ratios for GNRI (higher vs. lower GNRI/highest vs. lowest category, as reported); (**B**) pooled odds ratios for CONUT (higher vs. lower CONUT, as reported) [13,15,16,18,19,21,24,25].

In terms of continuous nutritional indices, the PNI demonstrated a consistent protective effect against sepsis mortality across a large population (N = 18,084). Higher PNI levels were associated with significantly lower odds of death (OR 0.64, 95% CI 0.50–0.83) and a reduced risk in survival analysis (HR 0.66, 95% CI 0.54–0.81) (Figure 4). For the GNRI, five studies confirmed that higher scores correlate with improved survival, resulting in a pooled HR of 0.66 (95% CI 0.44–0.98) (Figure 5A). In contrast, while the CONUT score suggested an increased risk of mortality for patients with higher scores (poorer nutrition), the aggregate random-effects estimate did not reach statistical significance (OR 1.83, 95% CI 0.94–3.54) (Figure 5B), likely due to the extreme heterogeneity (I^2^ = 97.4%) observed among the included studies.

#### 3.2.3. Comparative Prognostic Accuracy

**Figure 6:**
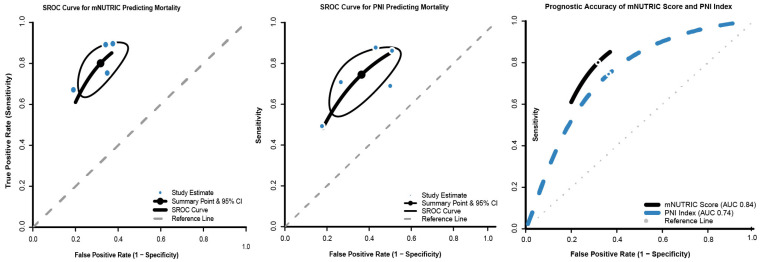
Summary receiver operating characteristic curves for mNUTRIC and PNI in predicting sepsis mortality.

In the diagnostic accuracy meta-analysis, mNUTRIC showed higher pooled discrimination metrics than PNI within their respective evidence bases (AUC 0.84 vs. 0.74). However, this represents an indirect comparison across separate study sets with heterogeneous thresholds, endpoints, and case mix and should not be interpreted as definitive superiority. The pooled SROC curve yielded an AUC of 0.84 (95% CI: 0.81–0.87) for mNUTRIC, which was substantially higher than the 0.74 (95% CI: 0.70–0.78) observed for PNI. Specifically, mNUTRIC achieved a summary sensitivity of 85.3% and specificity of 69.2%, whereas PNI showed more moderate performance (sensitivity 76.2% and specificity 58.4%) (Figure 6).

Fagan nomogram analysis further confirmed the clinical utility of mNUTRIC in sepsis risk stratification. Assuming a 30% baseline mortality risk, a high mNUTRIC score (PLR 2.77) increased the post-test probability to 54%, while a low score (NLR 0.21) effectively reduced it to 8%, providing more substantial added value for bedside decision-making than PNI (Figure S1). Importantly, the pooled OR/HR estimates reflect prognostic association, whereas SROC/AUC values summarize discrimination; these domains are complementary but not interchangeable.

#### 3.2.4. Subgroup and Heterogeneity Analysis

**Figure 7:**
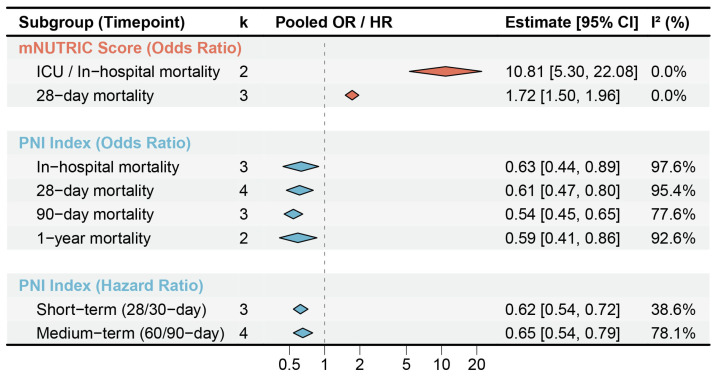
Time-stratified subgroup analyses of mNUTRIC and PNI in relation to sepsis mortality.

To explore potential sources of heterogeneity in the primary analysis, prespecified subgroup analyses were performed according to follow-up duration (Figure 7). For mNUTRIC, heterogeneity was substantially reduced after time-based stratification, with I^2^ decreasing from 84.0% to 0.0%. The pooled OR was 1.72 (95% CI 1.50–1.96) for 28-day mortality, whereas the pooled OR for ICU/in-hospital mortality was 10.81 (95% CI 5.30–22.08) (Figure S2).

For PNI, the protective association remained directionally consistent across different follow-up windows despite persistent heterogeneity. The pooled ORs were 0.63 (95% CI 0.44–0.89) for in-hospital mortality, 0.61 (95% CI 0.47–0.80) for 28-day mortality, 0.54 (95% CI 0.45–0.65) for 90-day mortality, and 0.59 (95% CI 0.41–0.86) for 1-year mortality (Figure S3A). A similar pattern was observed in the HR-based analyses, with pooled HRs of 0.62 (95% CI 0.54–0.72) for short-term survival and 0.65 (95% CI 0.54–0.79) for medium-term survival (Figure S3B).

### 3.3. Sensitivity Analysis and Quality Assessment

Leave-one-out analyses did not materially change the direction of pooled estimates, supporting the robustness of the main associations; detailed results are provided in Figure S4. The systematic exclusion of any single study, including large-scale cohorts such as Baek (2024) or Pan (2025) [16,23], did not significantly alter the direction or statistical significance of the primary pooled ORs and HRs (Figure S4). Specifically, for the mNUTRIC score, the pooled risk associations for both 28-day and ICU mortality remained stable and highly significant (*p* < 0.05) in all iterations, despite the exclusion of individual datasets. Formal assessment of publication bias using funnel plots or Egger’s test was omitted, as the number of studies for each specific nutritional index was below the recommended threshold of 10.

## 4. Discussion

Our meta-analysis provides an integrated view of the prognostic relevance of nutritional indices in sepsis. Three main findings emerged. First, elevated mNUTRIC and NUTRIC scores were consistently associated with increased mortality risk, and mNUTRIC showed the strongest overall discriminatory performance in diagnostic accuracy analyses. Second, higher PNI and GNRI values were associated with more favorable survival, supporting the concept that preserved nutritional and immunological reserve may be linked to better outcomes in sepsis. Third, the prognostic effects of these indices were not uniform across studies, and a substantial proportion of the observed heterogeneity appeared to be related to differences in mortality endpoints and follow-up duration. Taken together, these findings suggest that nutritional indices are not interchangeable instruments but rather reflect partially distinct dimensions of vulnerability in patients with sepsis.

The stronger prognostic performance of mNUTRIC and NUTRIC in this analysis is clinically plausible. Unlike conventional nutritional indices that primarily reflect baseline nutritional or immunological status, mNUTRIC and NUTRIC were developed specifically for critically ill patients and incorporate variables such as APACHE II, SOFA score, comorbidities, and time from hospital to ICU admission, which are closely linked to acute illness severity and underlying vulnerability in the ICU setting [9,31]. This broader construct likely explains why these scores showed a clearer association with sepsis mortality and why mNUTRIC, in particular, demonstrated good overall discrimination in diagnostic accuracy analyses across sepsis and other critically ill populations [3,14,32]. In patients with sepsis, where acute physiological derangement, organ dysfunction, systemic inflammation, and pre-existing comorbidity interact over a short time frame, a score that captures both nutritional risk and critical illness burden may be more closely aligned with near-term prognosis than indices based mainly on static laboratory nutritional markers [33].

At the same time, this apparent advantage should be interpreted with caution. The stronger performance of mNUTRIC does not imply that it is a purer measure of nutritional status or that it can be regarded as a nutrition-specific predictor in isolation. Its prognostic value may partly arise from the integration of established severity markers and comorbidity burden, which behave similarly to general ICU prognostic scores [31,34]. In this sense, mNUTRIC may function less as a narrow nutritional tool and more as a composite vulnerability score that performs well in ICU-based sepsis cohorts and other high-risk settings [14]. This distinction is important because it suggests that the superiority of mNUTRIC over simpler indices such as PNI, GNRI, or CONUT may primarily reflect differences in score design and intended use, rather than a straightforward hierarchy of nutritional assessment tools.

In contrast to mNUTRIC and NUTRIC, PNI, GNRI, and CONUT appear to capture a different layer of prognostic information. Rather than mirroring the global burden of acute critical illness, these indices are more closely linked to the baseline nutritional state, immune competence, and physiological reserve [35,36]. This distinction may help explain why higher PNI and GNRI values were generally associated with better survival in our analysis. In patients with sepsis, preserved albumin concentrations, lymphocyte count, body-weight-related reserve, and broader nutritional stability likely indicate a greater capacity to tolerate systemic inflammation, maintain organ function, and recover from prolonged catabolic stress [37,38]. From this perspective, these scores may be particularly useful as markers of host condition rather than as direct surrogates of acute disease severity [3].

Among the three, PNI showed the most consistent signal, with both pooled OR and HR estimates supporting a protective association. This stability is biologically plausible, as albumin and lymphocyte count are routinely measured and widely recognized as integrative indicators of nutritional and immune status across medical and surgical populations [39]. GNRI also demonstrated a protective association, although the evidence base was smaller and predominantly derived from survival analyses; prior work suggests its performance may be especially relevant in older or frailer patients, for whom nutritional and weight-related reserves may play a larger role in shaping clinical outcomes [40,41]. By contrast, CONUT showed a directionally unfavorable association with mortality, but the pooled estimate was not statistically significant and was accompanied by extreme heterogeneity. Existing data from medical, cardiovascular, and oncologic cohorts consistently link higher CONUT scores with worse prognosis [42,43], yet findings in sepsis remain inconsistent. The current evidence for CONUT in sepsis should therefore be regarded as insufficient rather than negative, and its role in sepsis prognostication remains uncertain pending larger, dedicated validation studies [44].

An important finding of this study was that a substantial proportion of the between-study heterogeneity appeared to be driven by differences in outcome definition and follow-up duration. For mNUTRIC, stratification by mortality endpoint markedly reduced heterogeneity, suggesting that these studies were not simply inconsistent but were assessing related scores against clinically different outcomes. ICU or in-hospital mortality is more closely linked to acute physiological deterioration and early treatment response, whereas 28- or 30-day mortality may already reflect a broader range of influences, including subsequent complications and recovery trajectory [31,45,46]. This likely explains why the strength of association varied across endpoint categories.

A similar consideration applies to nutrition-related indices evaluated over longer follow-up periods. The subgroup findings for PNI, together with the longitudinal patterns described for nutritional and inflammatory scores in critical illness and heart failure, suggest that the prognostic effect of baseline nutritional status may attenuate over time as post-discharge events, comorbidities, and evolving organ dysfunction increasingly shape risk [22,47,48]. Later outcomes are thus determined by many factors beyond the initial ICU presentation, and this should be taken into account when interpreting pooled estimates across heterogeneous time horizons. Overall, these findings suggest that the prognostic value of nutritional indices in sepsis is context-dependent rather than fixed and that comparisons across studies are most informative when outcome windows are aligned.

From a clinical perspective, these findings suggest that nutritional indices may provide useful adjunctive information for risk stratification in sepsis, as they are inexpensive, readily available, and based on routine clinical data. Among the evaluated tools, mNUTRIC appears to be more relevant to short-term prognostic assessment in ICU-based sepsis, whereas PNI and GNRI may offer complementary information on baseline nutritional and immunological reserve.

However, these indices should not be considered stand-alone prognostic tools or substitutes for established severity assessment. Their value is more likely to lie in complementing existing clinical judgment and conventional risk models, a role that still requires prospective validation.

## 5. Limitations

Several limitations warrant emphasis. First, most included studies were observational and predominantly retrospective, raising risks of residual confounding and selection bias, and adjustment sets were not standardized across studies. Second, heterogeneous thresholds/categorizations and variable mortality endpoints and follow-up windows limit the clinical interpretability of pooled categorical effects as a single actionable cutoff and contribute to substantial heterogeneity. Third, the compared tools are not construct-equivalent: mNUTRIC/NUTRIC incorporate illness-severity domains and may function as composite vulnerability scores, whereas PNI/GNRI/CONUT primarily reflect immunonutritional reserve; therefore, indirect comparisons cannot establish a definitive hierarchy. Fourth, although we cross-checked database-derived cohorts to minimize overlap, residual duplication cannot be fully excluded. Fifth, the included studies were concentrated in ICU settings and largely from Asian populations, which may limit generalizability. Finally, few studies assessed the incremental prognostic value beyond established sepsis severity scores or evaluated calibration/reclassification, and prospective head-to-head validation remains needed.

## 6. Conclusions

In summary, this meta-analysis suggests that nutritional indices are associated with mortality risk in patients with sepsis, although their prognostic performance varies across tools and clinical contexts. mNUTRIC and NUTRIC were more strongly associated with short-term mortality in ICU-based populations, whereas PNI and GNRI appeared to reflect a protective association consistent with better nutritional and immunological reserve. These findings indicate that nutrition-related indices may provide useful adjunctive information for prognostic assessment in sepsis, but they should not be interpreted as interchangeable or stand-alone predictors. Further prospective studies are needed to clarify their incremental value and to define how they can be most appropriately integrated into routine sepsis risk stratification.

## Figures and Tables

**Table 1:** Baseline characteristics of studies included in the meta-analysis.

Study (Year)	Country	Design	Sample Size (N)	Age, Years	Male (%)	Mortality Outcome	Threshold/ Category Definition	Indices Evaluated
Jeong (2018) [9]	Korea	Retrospective	482	66 (IQR: 56–75)	68.0%	28-day mortality	6	mNUTRIC, NUTRIC
Tsai (2021) [10]	China	Retrospective	1291	67.1	59.5%	7-, 14-, 28-, and 90-day mortality	6	mNUTRIC, NUTRIC
Hai (2022) [11]	Vietnam	Prospective	194	69 (59–80)	73.7%	In-hospital mortality	5	mNUTRIC
Wu (2022) [12]	China	Retrospective	2669	66.2 ± 16.5	55.6%	30-, 90-, and 365-day mortality	29.3	PNI
Wełna (2023) [14]	Poland	Prospective	146	66 (IQR: 58–74)	65.0%	28-day mortality	6	mNUTRIC
Yamaguchi (2023) [15]	Japan	Retrospective	307	74.7 ± 14.4	39.4%	ICU mortality	9	CONUT
Li (2023) [13]	China	Retrospective	2834	76.8	55.9%	28-day mortality	<82; 82–92; 92–98; ≥98	GNRI
Yildirim (2024) [17]	Turkey	Prospective	78	77.2 ± 9.9	46.2%	ICU mortality	6	mNUTRIC
Baek (2024) [16]	Korea	Retrospective	9763	74 (62–81)	58.2%	30-day mortality	CONUT: 0–1, 2–4, 5–8, 9–12; PNI: >38; 35–38; <35	CONUT, PNI
Calili (2025) [19]	Turkey	Prospective	126	66.5 ± 11.2	46.8%	In-hospital mortality	6	NUTRIC, NRS-2002, CONUT, PNI, and GNRI
Fang (2025) [21]	China	Retrospective	1649	NA	60.2%	60-day mortality	PNI: Q1 ≤ 30, Q2 30–37.7, Q3 > 37.7; GNRI: Q1 ≤ 95.5, Q2 95.5–111.9, Q3 > 111.9	PNI, GNRI
Cai (2025) [18]	China	Retrospective	4515	76.0 (70.0–82.0)	56.7%	28-day mortality	Q1: <78.92; Q2: 78.92–84.88; Q3: 84.88–90.84; Q4: >90.84	GNRI
Ren (2025) [24]	China	Retrospective	773	63.6	61.8%	28- and 90-day mortality	T1: <77.3; T2: 77.3–87.2; T3: ≥87.2	GNRI
Damarsoy (2025) [20]	Turkey	Prospective	115	77.6 ± 11.8	50.4%	In-hospital mortality	≤37.4	PNI
Pan (2025) [23]	China	Retrospective	6234	66.0 (18.0–95.0)	57.9%	14-, 28-, and 90-day mortality	T1: 1.30–6.45; T2: 6.50–9.75; T3: >9.75	PNI
Kollu (2025) [22]	Turkey	Retrospective	400	73 (18–95)	51.6%	ICU mortality	29.7	PNI
Xie (2025) [26]	China	Prospective	245	61.3 ± 16.1	73.5%	ICU mortality	6	NUTRIC
Xie (2025) [27]	China	Retrospective	5786	63.8 ± 16.7	56.4%	In-hospital, 28-, 90-, and 365-day mortality	<27; 27–33; ≥33	PNI
Shao (2026) [25]	China	Retrospective	2230	77 (65–102)	59.1%	28-day mortality	≤82; 82–92; 92–98; ≥98	GNRI
Wang (2026) [29]	China	Retrospective	7380	66	59.1%	28-day mortality	85	GNRI
Yang (2026) [28]	China	Retrospective	1350	NA	69.5%	28-day mortality	288.4	PNI
Zhao (2026) [30]	China	Retrospective	3202	71.0 (59.0–81.0)	56.3%	28-day mortality	34	PNI

**Abbreviations:** CONUT, controlling nutritional status; GNRI, Geriatric Nutritional Risk Index; ICU, intensive care unit; IQR, interquartile range; mNUTRIC, modified Nutrition Risk in Critically Ill; NA, not available; NUTRIC, Nutrition Risk in Critically Ill; PNI, Prognostic Nutritional Index.

## Data Availability

All data analyzed in this study were extracted from published articles. Further details are available from the corresponding author upon reasonable request.

## Supplementary Materials

The following supporting information can be downloaded at: https://www.mdpi.com/article/10.3390/jcm15103885/s1, Table S1: PRISMA 2020 Checklist; Table S2. Search strategies for each database; Table S3. Newcastle–Ottawa Scale assessment of included studies; Figure S1. Fagan nomograms for the clinical utility of A: mNUTRIC and B: PNI in predicting sepsis mortality; Figure S2. Time-stratified subgroup analysis of mNUTRIC according to mortality endpoint; Figure S3. (A). Forest plot of time-stratified subgroup analysis of PNI based on pooled odds ratios; (B). Forest plot of time-stratified subgroup analysis of PNI based on pooled hazard ratios; Figure S4. Leave-one-out sensitivity analyses of pooled prognostic estimates for nutrition-related indices and scores.

## Abbreviations

APACHE II	Acute Physiology and Chronic Health Evaluation II
AUC	Area Under the Curve
BMI	Body Mass Index
CI	Confidence Interval
CONUT	Controlling Nutritional Status
GLIM	Global Leadership Initiative on Malnutrition
GNRI	Geriatric Nutritional Risk Index
HR	Hazard Ratio
ICU	Intensive Care Unit
IL-6	Interleukin-6
MIMIC	Medical Information Mart for Intensive Care
MIMIC-IV	Medical Information Mart for Intensive Care IV
mNUTRIC	Modified Nutrition Risk in the Critically Ill
MNA	Mini Nutritional Assessment
MUST	Malnutrition Universal Screening Tool
NLR	Negative Likelihood Ratio
NOS	Newcastle–Ottawa Scale
NRS-2002	Nutritional Risk Screening 2002
NUTRIC	Nutrition Risk in the Critically Ill
OR	Odds Ratio
PLR	Positive Likelihood Ratio
PNI	Prognostic Nutritional Index
PRISMA	Preferred Reporting Items for Systematic Reviews and Meta-Analyses
REML	Restricted Maximum Likelihood
SAPS 2	Simplified Acute Physiology Score II
SGA	Subjective Global Assessment
SOFA	Sequential Organ Failure Assessment
SROC	Summary Receiver Operating Characteristic
